# Co-creating a program theory and evaluability assessment for an Irish single-session, synchronous chat-based youth mental health intervention: implications for outcome evaluation

**DOI:** 10.3389/fdgth.2026.1673317

**Published:** 2026-03-17

**Authors:** Maria Tibbs, Maeve Dwan-O’Reilly, Alexis Carey, Jeff Moore, Amanda Fitzgerald

**Affiliations:** 1 Youth Mental Health Lab, School of Psychology, University College Dublin, Bellfield, Dublin; 2 School of Psychology, University of Galway, Galway, Ireland; 3 Rehab Care, Dublin, Ireland; 4 Research and Evaluation, Jigsaw the National Center for Youth Mental Health, Dublin, Ireland

**Keywords:** digital mental health, implementation science, program theory, single-session interventions, synchronous chat-based support, youth mental health

## Abstract

**Introduction:**

Single-session online synchronous chat offers immediate, anonymous, single-session support for young people. However, the drop-in format attracts a diverse population with urgent and varied needs, creating challenges for evaluation. Standardized outcome measures may not capture short-term changes, and randomized controlled trials may be ethically inappropriate. These constraints point to the value of theory-based evaluation approaches rooted in implementation science, which can better capture short-term change, contextual complexity, and real-world variation in service delivery.

**Methods:**

This study applied a theory-driven evaluability assessment to Jigsaw Live Chat, an Irish single-session, synchronous chat-based mental health service for youth. A situational analysis, review of the literature, and an internal data review established the intervention context and examined existing evaluative approaches within the literature. Two co-design workshops with staff (*N* = 13) identified contextual barriers, preconditions and assumptions for intervention effectiveness, core components, mechanisms of change, and intended outcomes. These elements were synthesized into a program theory, which informed the development of recommendations for evaluation.

**Results:**

Several contextual factors were identified as crucial to the success of Jigsaw Live Chat, including cross-collaboration, integration, and buy-in across youth mental health services. Staff defined a range of core components (*N* = 12) of the intervention, detailing how these components might influence various outcomes such as accessibility, ease of use, perceived usefulness, help-seeking intention, and decreases in immediate distress and overwhelm among young people. The broader impacts of the service were developed, reflecting the potential contribution of synchronous chat within a wider, integrated youth mental health care system nationally. These findings were integrated into a causal program theory model, and tailored indicators were proposed to support feasible and meaningful evaluation.

**Discussion:**

This study presents one of the first published examples of an iterative, collaborative evaluability assessment of a single-session synchronous chat-based mental health support service. The findings clarify how such interventions operate within specific organizational and sociocultural contexts and provide the foundation for future adaptation and evaluation aimed at improving service delivery and outcomes for young people.

## 1 Introduction

Globally, youth mental health services are under pressure, with demand consistently outpacing capacity (1). This trend is particularly evident within the Irish context, where long wait times reflect ongoing capacity constraints in youth mental health services (2, 3), highlighting the urgent need for immediate, accessible support. In response, there has been a global shift towards developing and implementing youth-focused digital mental health services, especially online synchronous chat-based interventions. These services offer real-time, text-based support, typically in single sessions, via purpose-built platforms. They allow for immediate, interactive exchanges within a structured session and may be delivered by mental health professionals, trained volunteers, peer supporters, or AI-powered chatbots (4, 5).

Services such as Kooth (UK), eHeadspace (Australia), Kids Helpline (Australia), Kids Help Phone (Canada), Salerno (US), and Jigsaw Live Chat (Ireland) have become key providers of synchronous online chat support for young people. These interventions are widely promoted as low-cost and accessible alternatives to traditional support, yet evidence of effectiveness remains mixed, in part because the methodological tools used to evaluate them are often poorly aligned with their design. Much of the existing literature relies on trait-like symptom measures that require multi-week test–retest intervals and capture relatively stable characteristics [e.g., GAD-7 (6); RCADS (7) and transdiagnostic [e.g., SDQ (8), YP-CORE (9), K-10 (10)]. These measures are ill-suited to single-session chat support, where any change is likely to be immediate, situational, and state-like (11, 12). Nevertheless, many published evaluations continue to employ these instruments, assessing outcomes such as psychological distress, depression, anxiety, hope, or life satisfaction (13–17).

These difficulties are compounded by the diverse reasons young people use online synchronous chat support make measuring and testing effectiveness challenging. Operating on a drop-in basis, often without formal referral or screening, these platforms attract users with a broad spectrum of needs (73). Some seek informational support, while others face complex mental health concerns, often in moments of acute distress requiring immediate intervention (12–14, 75). This creates a heterogeneous group, making it difficult for standardized outcome measures to capture the full complexity of the service's impact (75). Moreover, ethical and methodological challenges also limit the feasibility and value of randomized controlled trials in this context. Withholding timely support from control groups may be inappropriate, and the core features of these services, such as anonymity and immediacy, are difficult to reproduce in controlled research environments. This reduces both the practicality of trials as well as the generalizability of their findings.

In response to this variability in profile, clinicians have reported adopting flexibility in their delivery of mental health support depending on the presenting need (4). Staff also frequently extend their role beyond direct support provision, including tasks such as information provision, referral to other services, assessment, and crisis management (4, 18). Accounting for these differences, it is perhaps unsurprising that adherence to specific procedures within these interventions is reported to be low. For instance, in an investigation of the activities within the online synchronous chat service offered by KidsHelpline, authors reported that only 52.9% of chats examined adhered to their pre-determined framework of therapeutic activities (19).

Reflecting this complexity, findings from outcome evaluations are mixed (20). Some studies report reductions in depression or psychological distress, although these improvements often do not exceed those observed in comparison conditions such as waitlist or treatment-as-usual controls (15, 16, 21). Similarly, studies examining outcomes such as life satisfaction and hope have reported limited or no change from pre- to post-intervention (14). Still, when young people are asked about their perceptions of effectiveness and overall service satisfaction, findings are promising, with many young people reporting feeling heard and understood (22–24). For example, Gould and colleagues found that while most young people did not report feeling more hopeful, less depressed, or less overwhelmed following the session, they nevertheless perceived the interaction as helpful and supportive (22). These findings highlight a common challenge in evaluating complex, brief interventions such as synchronous chat-based support, namely that standardized psychological outcome measures may not adequately capture the immediate, relational, or contextual benefits valued by young people. This measurement misalignment may contribute to apparently inconsistent evidence of impact, particularly when combined with heterogeneity in presenting needs and resulting variation in intervention delivery.

### 1.1 Complex interventions and their evaluation

The Medical Research Council (MRC) defines complex interventions as those involving multiple interacting components that target diverse behaviors and outcomes, require various skills from providers, are delivered across different groups and settings, and allow for flexibility in delivery (25). Online synchronous chat-based interventions meet these criteria due to the diversity of user presentations, flexible delivery, and challenges in establishing and measuring outcomes (26, 27).

Although RCTs were once seen as the “gold standard” for evaluating interventions, recent MRC guidance recognizes their limitations in capturing real-world complexity (25). This shift aligns with the emergence of implementation science, which goes beyond merely establishing intervention effectiveness to address the reality that such effectiveness does not ensure routine adoption in applied settings (28). Implementation science increasingly prioritizes the translation and integration of scientific evidence into practical applications and real-world settings, emphasizing the need to consider contextual barriers and facilitators that affect the uptake of these interventions (29, 30).

Furthermore, given the tightly controlled and experimental nature of randomized trials, they cannot always capture how an intervention works or whether it is scalable and transferable to different settings. Even when RCTs demonstrate a significant effect, implementation science is essential to understanding *how* and *why* the intervention works and the mechanisms underpinning its success (31). Conversely, if an intervention shows no effect, theory-informed approaches such as program theory are critical for identifying which components may need to be adapted or improved. In recognition of this, the MRC now endorses theory-based evaluations, such as evaluability assessments, and the development of program theory to ensure findings are relevant and applicable. This approach is essential for accurately addressing the complexities inherent in real-world interventions (25). In line with these implementation science approaches to evaluation, a range of frameworks have been developed to guide the planning and evaluation of community-based and applied interventions.

### 1.2 Evaluability assessments

The latest MRC guidelines recommend using evaluability assessments (EA) to determine if an intervention is ready for evaluation and how best to conduct it (25). These structured, participatory processes involve stakeholders in clarifying the intervention's aims, components, and expected outcomes, while generating practical, context-specific recommendations for evaluation. By involving stakeholders throughout, EAs foster consensus, build evaluation capacity, and ensure alignment with service needs (18, 19).

EAs follow a structured process that includes a combination of deductive and inductive methods. Deductive approaches can include the review of internal service/intervention documents and an examination of relevant literature to describe the intervention and assess the influence of both national and organizational contexts, whereas inductive methods generally include the co-design of an intervention program theory (26, 32). Sequentially, the steps included in this process are:

1. Engaging stakeholders from the outset
2. Assessing the focus or goal of the evaluability assessment
3. Reviewing existing literature and internal data sources
4. Co-developing a program theory
5. Making recommendations for evaluation based on the evaluability findings (32, 33).

Co-producing a program theory is central to all EAs (32–35). A program theory explains how an intervention is expected to achieve its intended outcomes. A well-defined program theory is crucial for comprehending how an intervention operates; without it, the intervention may be perceived as a *black box* (26), hindering a clear understanding of its underlying mechanisms and outcomes. According to the MRC, program theories should outline the core components of the intervention, their interactions with one another, the mechanisms of change resulting from these components, the resulting outcomes, and the real-world impact of the intervention. These elements are typically illustrated in the form of a theory of change model, logic model, or systems map (28, 36). For a more detailed description of the program theory terminology, see Supplementary Materials.

### 1.3 The importance of capturing intervention “context”

Implementation science and realist evaluation approaches highlight that change happens through a complex interaction of context, mechanisms, and outcomes [CMO; (29, 37)]. To understand how an intervention works in real-world settings, it is crucial to consider the context in which it operates. Therefore, as recommended by the MRC, program theories should include the key contextual factors and implementation assumptions that influence how the intervention leads to behavior change (25).

Context refers to any external factors that influence the success of an intervention (38) and has become a key focus in implementation science evaluation approaches. Specifically, context encompasses a range of social, cultural, economic, and environmental factors that shape how the intervention operates. Craig and Campbell (33) emphasize the need to consider context throughout the entire lifecycle of an intervention, from development to evaluation. Addressing context early can enhance an intervention's effectiveness, appropriateness, and sustainability, and helps clarify why an intervention may succeed in one setting but fail in another, potentially excluding certain groups and contributing further to health or mental health inequalities.

### 1.4 The current study

Despite the growing use of online synchronous chat interventions for young people, there is limited published work applying evaluability assessment and theory-driven approaches to theorize how and why these interventions work in practice. Given the complexity of anonymous, chat-based support, and the challenges associated with evaluating brief, single-session interventions, it is essential to understand how they are designed to work in practice. To date, only a small number of published program theories have been developed for youth-focused online support services, with existing examples largely centered on multi-component or multi-session interventions, such as Kooth in the UK (39). As such, there remains a gap with respect to theory-driven models and evaluability assessments focused specifically on single-session, synchronous chat-based mental health interventions for young people.

The present study addresses this gap by developing a program theory and evaluability assessment of an Irish online synchronous chat-based service, Jigsaw Live Chat. While the service captures demographic and help-seeking information, along with pre-session psychological distress measures [YP-CORE for those aged 12–16 (7) and CORE-10 for those aged 17+ (40)], and post session satisfaction [adapted from eHeadspace (41)], greater clarity was needed around its core aims, underlying mechanisms, and the most appropriate approaches to evaluation. Although program theories are ideally developed during implementation planning, they can also be constructed retrospectively to inform future implementation, evaluation, and sustainability (42, 43). Thus, this study undertook a retrospective evaluability assessment through the development of a program theory, guided by the following research questions:

1. How do staff members operationalize Jigsaw Live Chat?
2. What real-world impacts does Jigsaw Live Chat contribute to?
3. What outcomes does Jigsaw Live Chat seek to achieve?
4. How and under what circumstances does Jigsaw Live Chat achieve these outcomes?
5. Based on the theorizing of Jigsaw Live Chat, how might Jigsaw Live Chat be evaluated?

## 2 Materials and methods

### 2.1 The intervention

Jigsaw – National Center for Youth Mental Health, an Irish primary-care level in-person and video-based mental health support service for young people, launched its nationally available synchronous chat service, Jigsaw Live Chat, in 2020. Jigsaw Live Chat provides real-time, single-session, text-based mental health support with trained mental health professionals, including psychologists, psychotherapists, occupational therapists, social workers, and mental health nurses. While the intervention primarily follows person-centered and solution-focused approaches, clinicians may integrate cognitive behavioral therapy (CBT), emotion-focused therapy, and other modalities based on their expertise and the young person's presenting needs. Young people log onto the chat portal to access support and enter a waiting queue. Once a clinician becomes available (typically within three minutes), the session lasts approximately 45–50 min. For futher information on the profile of young people that attend this service, please see Tibbs et al. (75).

### 2.2 Procedure

In line with guidance from Trevisan and Walser (32), the current study adopted an iterative, multi-method, participatory evaluability assessment with four key stages (Focusing the evaluability assessment, developing an initial program theory, gathering feedback, using the evaluability assessment).

#### 2.2.1 Recruitment of intervention stakeholders

Participants were recruited through gatekeepers within the organization. Gatekeepers identified relevant participants according to a series of inclusion criteria provided by the primary researcher (have worked, either as operational or clinical staff, in live chat services with young people). Gatekeepers circulated an expression-of-interest email to *N* = 25 staff members, followed by one reminder from the research team. Fifteen staff volunteered to participate, yielding a 60% response rate, comprising 7 mental health clinicians, 4 managerial clinical staff, 2 managerial operational staff, and 1 service administrator. Due to scheduling conflicts, 40% attended both workshops, with 13 participants attending the first and 8 attending the second. To ensure all *N* = 15 staff had the opportunity to contribute, feedback was collected via email after each workshop. Summaries of the first workshop's findings were presented at the second, and a summary of the second workshop was sent to all participants for further feedback.

#### 2.2.2 Developing an initial program theory

To inform the development of the program theory, a desk-based situational analysis and internal data review were conducted. While the findings from these components are not reported in detail here, the situational analysis mapped the broader healthcare, policy and demographic context drawing on publicly available national reports and demographic information, while the internal data review examined user profiles, funding applications, the Live Chat platform and relevant reports to understand implementation, outcomes and gaps in measurement. The STRiDE brief situational analysis tool guided the contextual review [See Supplementary Materials for a copy of this checklist; (74)].

To co-develop the program theory, two virtual co-production workshops were conducted via Zoom® with participants. The Zoom “Whiteboard” was used to anonymously capture participants' perspectives via sticky notes. Pre-designed templates adopting program theory terminology (See Supplementary Materials for detailed definitions of this terminology) facilitated structured discussions, with each workshop lasting three hours, including a 20-minute break. To mitigate power dynamics, facilitators emphasized confidentiality and guided participants to anonymize their cursors.

The first workshop focused on defining the intervention, its impacts, and components, with participants choosing to contribute verbally or through virtual sticky notes. Facilitators summarized and grouped these responses live within the workshop and collated them afterward into a draft program theory model. The second workshop gathered feedback on this draft program theory model and refined the model asking participants to identify mechanisms of change, and participants reported contextual factors influencing the intervention's success.

#### 2.2.3 Gathering feedback on the program theory

**Figure 1:**
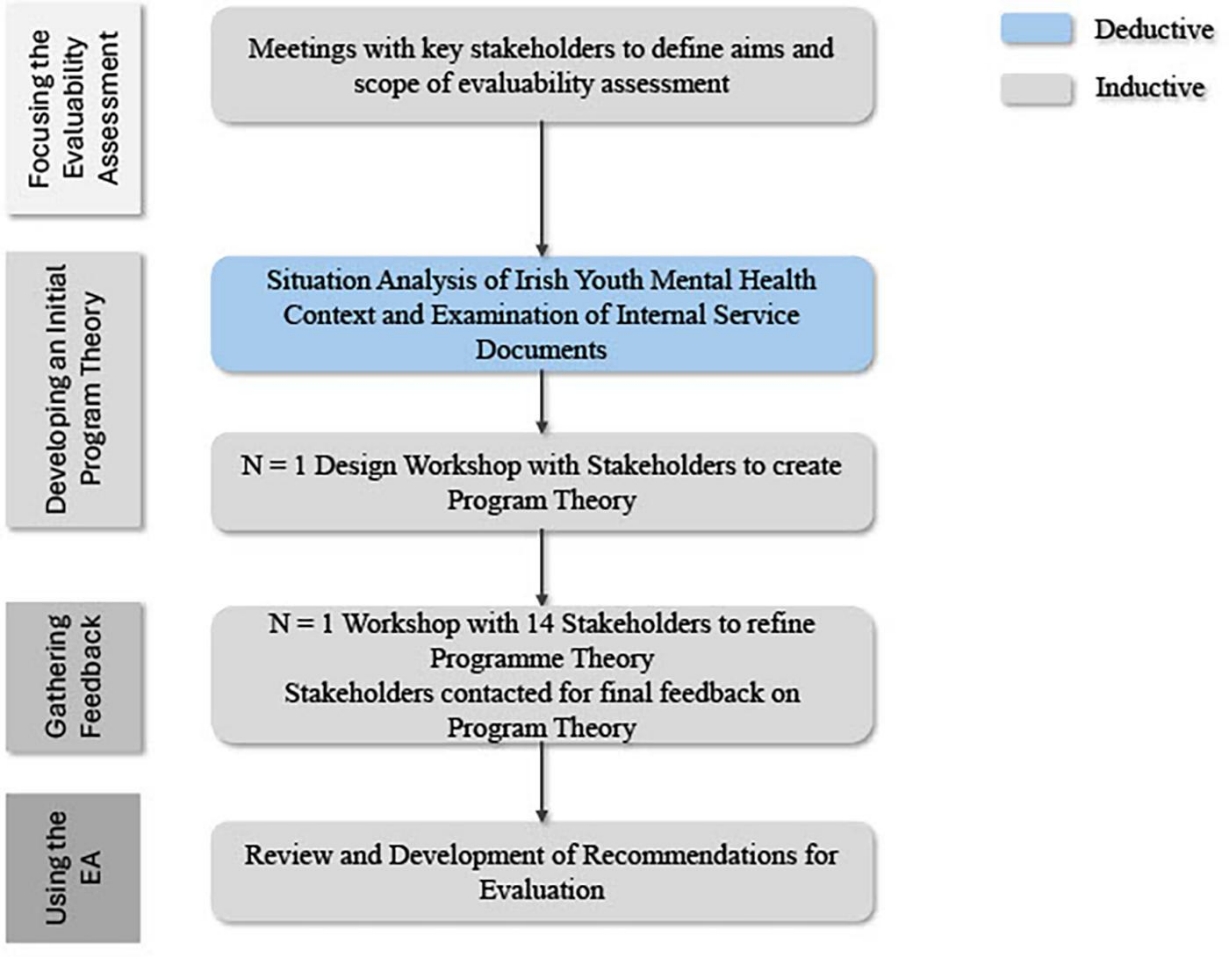
Visual summary of the evaluability assessment process. This diagram outlines the stages used to assess evaluability, including system context review, stakeholder engagement, and theory development.

Following the workshops, participants provided final feedback on the drafted program theory via email. A visual summary of the process is provided in Figure 1, and a list of questions posed by the researchers during the workshops can be found in the Supplementary Materials.

#### 2.2.4 Using the evaluability assessment

Recommendations for measurement and evaluation were developed through the situational analysis and program theory development, integrating identified contextual factors, intervention components, and mechanisms of change with national policy and governance documents, peer-reviewed research on youth mental health and digital interventions, and evidence from a preparatory systematic review of 18 comparable online chat services. These recommendations aimed to assess key outcomes, ensure feasibility within the constraints of a single-session chat context, and support ongoing service improvement.

While the program theory clarified how the intervention is currently delivered, the RE-AIM framework (44, 45) was used to structure analysis of the evaluability findings and guide recommendations for future monitoring and evaluation aligned with RE-AIM's five core dimensions: reach, effectiveness, adoption, implementation, and maintenance (44). The RE-AIM has been frequently adopted to guide the development of evaluation frameworks from an implementation science perspective (46).

### 2.3 Data analysis

Data were extracted and charted from all sources (situational analysis, internal service data and the iterative workshops according to the specific program theory terminology outlined in the Supplementary Materials). The development of the program theory was informed by an iterative analysis of the workshop data. Two researchers (MT; MDO'R) jointly reviewed and charted the workshop notes using the program theory terminology outlined in the Supplementary Materials, meeting to compare interpretations, refine constructs and map causal associations across emerging components. Differences in interpretation were resolved through discussion and by returning to the original workshop notes. The analysis was iterative, with provisional versions of the program theory developed after the first workshop, presented back to participants during the second workshop for member checking, and then refined further based on their feedback.

The situational analysis and internal service data were reviewed separately and used to contextualize and supplement the workshop findings rather than being analyzed as qualitative data. This combined process ensured that the program theory reflected both stakeholder perspectives and the broader contextual evidence base.

Evidence from the preparatory systematic review of 18 synchronous chat-based mental health interventions was similarly not subjected to formal data analysis within the program theory development. Rather, the review was used to situate the evaluability assessment within the wider international literature, support identification of plausible outcome domains, and inform the development of appropriate and feasible recommendations for measurement and evaluation within a single-session, anonymous chat context.

The Program Theory model was refined based on workshop data and facilitator notes, mapping causal associations across each construct. The online note-taking tool Notion® was used to collate and store information extracted from reports, policies, and census data. These data were included to provide contextual information on demographic patterns, levels of youth mental health need, current provision and likely demand for online support, which helped inform the assumptions and contextual factors identified in the situational analysis. These data, combined with workshop insights, informed the contextual factors associated with current and future implementation success.

Components of the intervention identified from the evaluability assessment were mapped to Behavior Change Techniques (47) to provide a strong theoretical basis for the components and to enhance the adaptability and replicability of the intervention. However, the BCT terminology was not incorporated into the final program theory to maintain consistency with Jigsaw's existing service delivery language and ensure alignment with stakeholder preferences.

### 2.4 Ethical approval

Ethical approval for the current study was obtained from the University College Dublin Human Research Ethics Committee [approval number: HS-LR-24-8-Tibbs-Fitzgerald] and Jigsaw's Research Ethics Committee [approval number: JREC-E-2023-005]. All participants provided informed consent to take part in the workshops. No personal information was collected from the participants, and workshops were not recorded to ensure confidentiality. Notes taken from the workshops were anonymized and stored in an encrypted file with access granted to collaborators on the project.

## 3 Results

Findings from the evaluability assessment are organized around the key constructs of the program theory, including intervention conceptualization, contextual factors, resources and preconditions, activities and components, mechanisms of change, outcomes, impact, and implementation assumptions. Collectively, these constructs address the study research questions by specifying how the intervention is operationalized in practice, how and under what circumstances change is expected to occur, and what outcomes and broader impacts the service seeks to contribute to. The section concludes with recommendations for evaluation and measurement.

### 3.1 Intervention conceptualization

Staff reported several key design and delivery features of Jigsaw Live Chat that described the intervention. They emphasized its accessibility, including national reach, the absence of formal referral processes, and minimal wait times. Attendees reported that the increased accessibility allowed young people greater agency and autonomy in their support and information-seeking. A key distinction noted by staff was that this support was delivered by trained mental health professionals, unlike other services staffed by volunteers or chatbots. Staff also highlighted that young people used the service for both emotional support and information and considered this dual purpose a defining feature. Finally, the “*in-the-moment*” nature of the service was reported as critical when describing the service, allowing young people to access support when needed, a design feature often absent in traditional youth mental health services. The following conceptualization was developed by participants during the first co-production workshop:

“Jigsaw Live Chat is a nationally available, anonymous, and professionally provided online chat service that offers low-barrier, compassionate, and autonomous single-session support to young people aged 12–25 who are seeking in-the-moment mental health support or information”.

### 3.2 Program theory model

**Figure 2:**
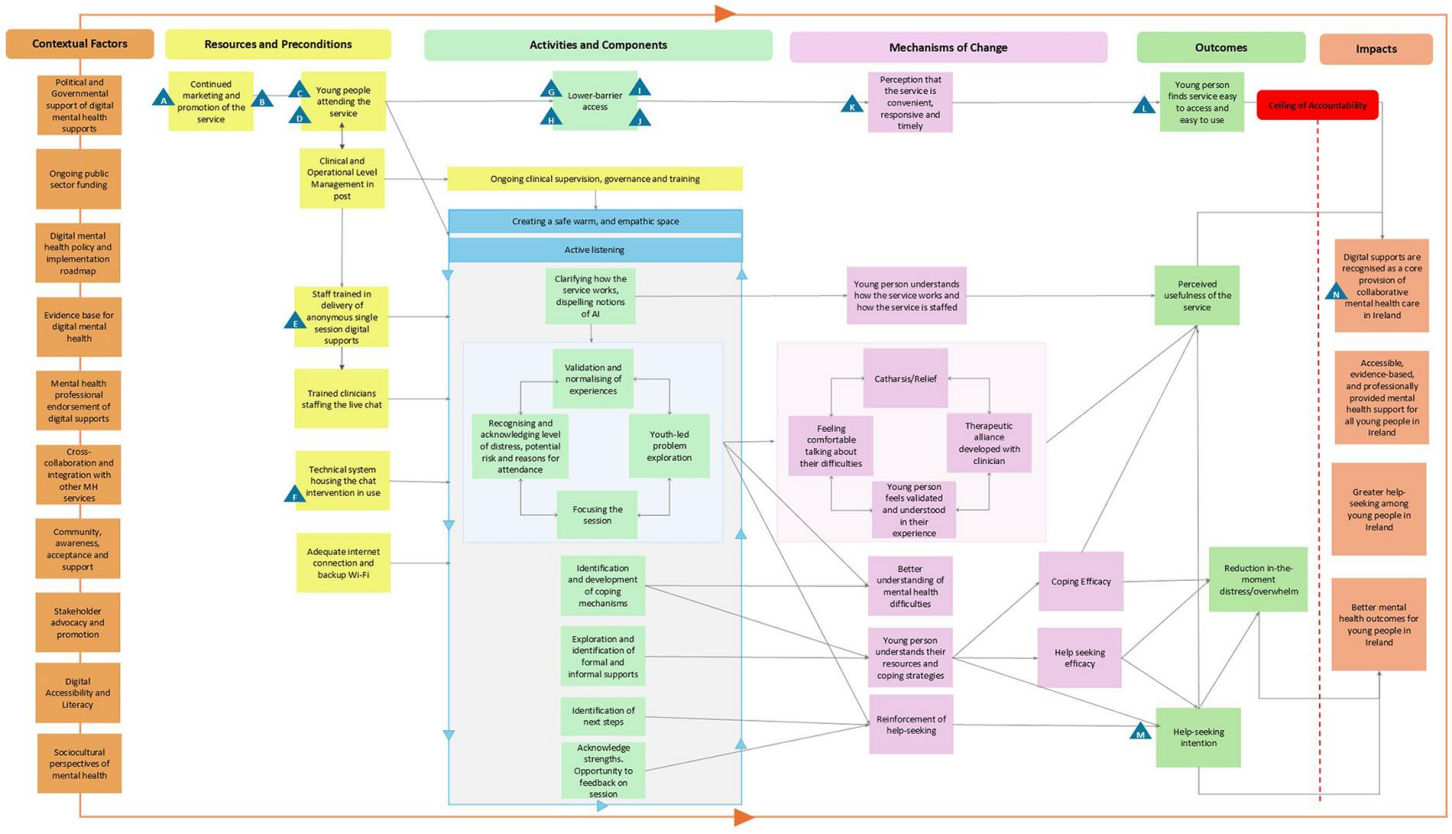
Program theory model of Jigsaw Live Chat. The model illustrates, from left to right, the contextual factors and resources necessary for delivery, the core intervention components, the mechanisms of change they activate, and the outcomes and wider impacts to which the intervention contributes. Triangles indicate implementation assumptions. Elements beyond the ceiling of accountability represent factors to which the intervention may contribute but which remain outside its direct control.

The Program Theory model depicts how the service functions and achieves change, structured across its seven core components. Arrows between the components represent how one element of the Program Theory Model leads to the next, for example, how intervention outcomes lead to impacts. Some arrows include proposed circular or bidirectional relationships, as denoted by double-sided arrows in the Program Theory Model. For a graphic depiction of this model, see Figure 2.

### 3.3 Contextual Factors

Four key national or “macro” level contextual factors were identified as crucial for the implementation of the Jigsaw Live Chat Intervention, grouped into four interdependent levels: 1) Governmental/Policy, 2) Mental Health System and Service Provision, 3) Research and Evidence, and 4) Community.

#### 3.3.1 Governmental level and policy factors

Securing governmental support for digital mental health interventions, both financially and politically, was deemed essential for the success of live chat. This includes continued public sector funding and political endorsement, which could be hindered by frequent changes in government. Staff also highlighted the need for a national digital mental health policy, alongside digital literacy training for young people, particularly in rural areas where broadband access is more limited.

#### 3.3.2 Mental health service provision

Staff stressed the importance of buy-in from mental health professionals for the adoption of digital tools like jigsaw live chat. Staff outlined potential skepticism about the effectiveness of digital support, which could hinder their integration into existing mental health services. Strong cross-collaboration between digital and traditional mental health services, particularly those provided by the public health bodies, was also seen as critical for effective service delivery and support for young people.

#### 3.3.3 Research and evidence

A robust evidence base for digital mental health interventions was highlighted as essential for securing buy-in at all levels. Demonstrating the effectiveness of digital tools could challenge misconceptions about their value compared to traditional in-person services.

#### 3.3.4 Community-level factors

Sociocultural attitudes towards mental health and digital mental health supports were identified as key to the success of the intervention. Negative perceptions of digital mental health, particularly among young people, parents, and educational institutions, could limit access. Community advocacy for digital support, particularly from parents, schools, and GPs, could enhance service reach and influence policy-level decisions on funding and provision.

The factors outlined in workshops were combined with those identified through the situational analysis of the Irish mental healthcare system. For a full, detailed description of these contextual factors, see Supplementary Materials.

### 3.4 Resources and preconditions

Several Jigsaw or internal organizational-related resources were highlighted as fundamental to the functioning of Jigsaw Live Chat. Specifically, ongoing marketing of the service and resulting attendance from young people were noted as fundamental to the basic operation of the service. Similarly, necessary for intervention operation were ongoing supervision of staff, clear clinical governance, guidelines, and training. Attendees highlighted the importance of training both in the delivery of the chat-based single-session support offered by Jigsaw Live Chat and the provision of ongoing, continual professional development to ensure the effective delivery of the intervention. Specifically, training to support staff in LGBTQ+-affirmative practice, crisis management, and single-session approaches were considered essential. Finally, to operate the service successfully, staff outlined the need for an adequate technical system to house the intervention, as well as continual technical management and upkeep of the system by dedicated developers. The necessity of a strong Wi-Fi connection was also noted. Ultimately, without staff, training, and technical infrastructure resources, the service could not reliably or successfully function both in the short and long term.

### 3.5 Intervention activities and components

A range of activities and intervention components were identified during the workshops and were considered crucial to achieving both the outcomes and impacts of the intervention. Aside from components specific to the design of the overall service, such as the lower-barrier accessibility due to the absence of formal referrals and shorter wait time than traditional services, these activities and components generally focused on rapport building with the young person and techniques to support them with their presenting problem.

#### 3.5.1 Creation of a virtual “space” and relationship building in the chat-based environment

Workshop attendees highlighted that creating a safe and warm empathic virtual “space” and practicing active listening were essential to the success of chat sessions. Although these foundational elements are intentionally established at the beginning of the session, they remain integral throughout, supporting other techniques used during the interaction. Many attendees emphasized the importance of “space” as a therapeutic concept, noting that a sense of safety can also be fostered in virtual chat settings through text-based techniques and rapport-building strategies.

Welcoming the young person was something clinicians regularly did at the start of the session to create this “space”. This often involved asking how the young person was. Increasing emphasis was placed on clarifying that the young person was speaking to a real person, not a chatbot. This involved explicit behaviors such as telling the young person that they were human or, more implicitly, using informal or colloquial language. Some clinicians also noted the deliberate use of imperfections or slight misspellings to better convey this. Similarly, staff noted the use of emojis between sentences or points. To further create this sense of “presence” and create a safe space for the young person, staff highlighted that they would often validate the young person in their attendance at the chat service, noting that young people attending sometimes reported feeling their issues were not serious enough or that they were taking up clinician time unnecessarily.

Staff emphasized the importance of using the young person's reasons for attendance and measures of psychological distress reported before the chat session as tools through which to ask focused questions about why they may have attended or to guide empathic commentary about how the young person might be feeling. They highlighted their use of paraphrasing through text, validating the young person in their messaging to show empathy, and continual “checking in” with the young person to further build rapport and to encourage open communication. They also detailed the importance of directly labelling emotions and using clarifying questions to minimize misinterpretation and to more clearly convey emotion, using statements such as “*I sense that..*”, and “*Do I have this right?*”.

#### 3.5.2 Therapeutic techniques and behaviors in the chat-based environment

While staff delivering the Jigsaw Live Chat service were multidisciplinary and adopted a range of tools specific to their training, there was a range of common techniques reported by workshop attendees that were used within the chat environment. Specifically, staff noted the importance of setting a focus or goal for the session. This was especially important as some young people may attend with many concerns that may not be possible to address within a single 50-minute session. This process was guided primarily by the young person, with the clinician supporting them to identify a realistic and meaningful goal. To support this, clinicians often drew on the young person's reason for attendance and their pre-session psychological distress scores as entry points for focused questioning, empathic commentary, or discussion about how the young person might be feeling. In addition, some staff outlined that they might ask the young person what their expectations for the session are and align this with what might be reasonably achieved within the session.

A core feature of the chat sessions was the use of a strengths-focused approach, including identifying coping strategies the young person had used successfully in the past. Staff also explored existing support networks and encouraged young people to seek additional support from family or friends. Similarly, they guided young people to additional services or answered questions related to help-seeking to encourage further support if needed. Exploring both formal and informal support was especially important for risk management, particularly when a young person presented with suicidal ideation or self-harming behaviors. In those cases, staff outlined that they would discuss passing information to emergency services and/or explore the potential for that young person to contact a crisis service, while simultaneously exploring any protective factors the young person might have.

Some staff described encouraging young people to elaborate on their immediate emotions or presenting problems through text during the session. They also noted the importance of giving the young person space to reflect and report these emotions by facilitating pauses. Staff discussed striking a balance between what could be considered helpful pauses vs. lapses in sessions. When pauses were lengthy, staff would check whether the young person was still present or offer gentle reminders that they were available to talk. Interestingly, staff noted that young people sometimes responded to pauses with unexpectedly direct comments, such as “*Why are you taking so long to respond?*”. This directness often continued throughout the session and was interpreted as a reflection of the emotional intensity and immediacy that can characterize chat-based communication. For clinicians, empathy and active listening were consistent features throughout the entirety of the chat session, with staff noting that they would use deliberate language to convey that they are listening, for example, “*I hear you*”, “*I’m here*”, and “*It sounds like….*”.

Toward the end of the session, staff stressed the importance of reminding the young person that the time was coming to an end. Checking in to assess how the young person was feeling, welcoming them to return if needed, or directing them to additional services were outlined as key features of this phase of the chat. In line with the strengths-focused approach taken by staff, they may reiterate the strength and courage of the young person to attend the chat session, remind the young person of their coping skills, and identify something that the young person can do immediately following the chat session. Staff noted that, depending on the level of distress and complexity, achieving a resolution was not always possible within a single-session chat format. However, in this case, checking in with the young person and reiterating strengths was noted as even more crucial.

#### 3.5.3 Mapping to behavior change techniques

A range of the techniques discussed by staff and those identified through the document review can be reliably mapped to techniques as described by the Behavior Change Taxonomy (47). From the taxonomy of *N* = 93 behavior change techniques, *N* = 10 could be identified as present within the Jigsaw Live Chat intervention. For a list of these behaviors with their corresponding BCT identification number, see Table 1.

**Table 1:** Behavior change techniques used in Jigsaw Live Chat intervention.

Behavior change technique	Definition (Michie et al., 2013)	Example within Jigsaw Live Chat intervention
1.1 Goal setting (behavior)	Set or agree on a goal defined in terms of the behavior to be achieved.	Depending on the collaboratively chosen focus of the session, identify a specific activity or behavior to work on.
1.2 Problem-solving	Analyze, or prompt the person to analyze, factors influencing the behavior and generate or select strategies that include overcoming barriers and/or increasing facilitators (includes “Relapse Prevention” and “Coping Planning”).	Guiding young people to additional informal and formal support. Identifying what has worked well in the past to help young people with current concerns.
1.3 Goal setting (outcome)	Set or agree on a goal defined in terms of a positive outcome of wanted behavior.	Depending on the collaboratively chosen focus of the session, identify a specific mental health concern to work on.
1.4 Action planning	Prompt detailed planning of performance of the behavior (must include at least one of context, frequency, duration, and intensity). Context may be environmental (physical or social) or internal (physical, emotional or cognitive) (includes “Implementation Intentions”	Identification of next steps immediately following the session.
1.5 Review outcome goal(s)	Review behavior goal(s) jointly with the person and consider modifying goal(s) or behavior change strategy in light of achievement. This may lead to re-setting the same goal, a small change in that goal, or setting a new goal instead of (or in addition to) the first, or no change.	Collaboratively working on the goal throughout the Live Chat session
3.2 Social support (practical)	Advise on, arrange, or provide practical help (e.g., from friends, relatives, colleagues, “buddies” or staff) for performance of the behavior.	Guiding young people to additional informal and formal support.
3.3 Social support (emotional)	Advise on, arrange, or provide emotional social support (e.g., from friends, relatives, colleagues, buddies or staff) for performance of the behavior.	Provision of multi-disciplinary mental health support from trained mental health professionals.
13.2 Framing/reframing	Suggest the deliberate adoption of a perspective or new perspective on behavior (e.g., its purpose) in order to change cognitions or emotions about performing the behavior (includes “Cognitive structuring”).	Adopting a strength-focused approach, encouraging the young person to recognize their strengths and coping skills.
13.4 Valued self-identity	Advise the person to write or complete rating scales about a cherished value or personal strength as a means of affirming the person's identity as part of a behavior change strategy (includes “Self-affirmation”).	Adopting a strength-focused approach, encouraging the young person to recognize their strengths and coping skills. Reiterate young people's courage for attending the service.
15.1 Verbal persuasion about capability	Tell the person that they can successfully perform the wanted behavior, arguing against self-doubts and asserting that they can and will succeed.	Adopting a strength-focused approach, encouraging the young person to recognize their strengths and coping skills. Reiterate young people's courage for attending the service.
15.3 Focus on past success	Advise to think about or list previous successes in performing the behavior (or parts of it).	Identifying what has worked well in the past to help young people with current concerns.

### 3.6 Mechanisms of change

Staff noted that youth-led exploration, including the identification of coping strategies and expressions of validation, helped young people better understand their presenting problems. This process appeared to enhance their sense of coping and awareness of available support. Staff also described how active listening, empathy, and validation contributed to the development of a brief but meaningful therapeutic alliance. Even within a single session, this alliance fostered emotional safety, enabling young people to engage more openly and reinforcing their confidence in seeking further help. For those seeking information or emotional support, recognizing both formal and informal resources may strengthen help-seeking confidence and perceived self-efficacy.

### 3.7 Outcomes

#### 3.7.1 Perception that the service is accessible and easy to use

The service is designed to reduce structural and stigma-related barriers commonly associated with attending traditional in-person services. If effective, young people should report that the service was easy to access, and that the platform was simple to navigate and use.

#### 3.7.2 Perception that the service is useful and meets young people's needs

By explaining how the service operates and setting clear expectations, young people are more likely to understand what can realistically be achieved within a single chat session. If successful, this should lead to perceptions that the service is useful and responsive to their needs. Within the session, this usefulness may be further enhanced through the development of a therapeutic alliance, open discussion about mental health, and the young person feeling validated and understood, all of which may contribute to fostering emotional relief. Since young people come to Jigsaw Live Chat for many different reasons, success may be reflected in progress toward collaboratively agreed-upon goals for that specific session. Some staff described expecting young people to feel more hopeful after the session. Across many definitions of hope, including those used in traditional psychological measures, a key component is a sense of agency, that is, feeling capable of making progress toward meaningful goals. This is a particularly relevant and important short-term outcome within the context of single-session anonymous chat-based support.

#### 3.7.3 Decreases in immediate psychological distress and feelings of overwhelm

Some staff reported that following the intervention, they expected young people to experience a reduction in the intensity of their psychological distress and to feel less overwhelmed or calmer. However, given the wide range of reasons young people attend these services, staff noted that distress reduction may not be a relevant or realistic outcome for all users. Some staff suggested that while immediate reductions might be modest, these small shifts could support improved wellbeing over time when combined with other support or repeated help-seeking.

#### 3.7.4 Help-seeking intentions

Given that many young people use Jigsaw Live Chat to seek information about mental health support, and that for some, it may be their first time engaging with any service, staff described the intervention as a potential 'steppingstone' toward further help-seeking. The person-centered and youth-friendly nature of the service was seen as especially important for those who might be hesitant to seek support or who hold negative perceptions of traditional services. The clinicians would often signpost young people to additional services, including Jigsaw services, including Jigsaw Live Chat. This combination of informational support and reinforcement of help-seeking could improve help-seeking efficacy and increase intentions among service users.

### 3.8 Intervention impacts

Following the first workshop, staff-identified impacts were refined using contextual insights from the situational analysis to ensure alignment with the broader Irish mental health landscape. Collaboratively, staff at the second workshop identified four relevant impacts for Jigsaw Live Chat.

#### 3.8.1 Digital support is recognized as core to the provision of collaborative mental health care

Staff emphasized that Jigsaw Live Chat is just one component of a broader mental health care system for young people in Ireland. They noted that there may be apprehensions, skepticism, and misconceptions about the benefits of digital support among mental health professionals, young people, and the public. However, staff highlighted that the positive outcomes of the intervention could foster change and lead to greater acceptance of digital services. Additionally, they recognized that if the service is perceived as useful and effective, and is promoted effectively, Jigsaw Live Chat could integrate into a collaborative system of mental health support, working alongside other services to enhance care for young people.

#### 3.8.2 Accessible, evidence-based, and professionally provided mental health support for young people

Staff noted that Jigsaw Live Chat could contribute to the delivery of more accessible support to young people, given its low-barrier design. Given the lengthy wait times and rural inequalities that impact many traditional services, Jigsaw Live Chat could serve as a key facilitator toward more accessible care for young people in Ireland. However, many stressed that the service ultimately could not be described as a “no-barrier” service as access remains uneven due to differences in literacy, digital skills, socioeconomic status, and physical impairments. However, the service could, if successful, contribute to a larger concerted effort toward accessible and professionally provided support to young people nationally.

#### 3.8.3 Greater help-seeking among young people

Many staff described Jigsaw Live Chat as a “steppingstone” to further care. Some young people used the service to seek help-seeking advice, others as a bridge between sessions, or as an initial entry point into the mental health system. If the service enhances help-seeking efficacy and intention, it may support more young people to independently seek appropriate mental health support.

#### 3.8.4 Better mental health outcomes for young people

Staff outlined their aspirations for the service to contribute to better mental health outcomes for young people. If a young person perceives the service as useful, increases their help-seeking intentions, and meets their needs, this could contribute to better mental health outcomes for young people nationally. In line with this, staff emphasized the importance of the service's role in onward help-seeking, suggesting that additional appropriate services could improve mental health outcomes for young people who used Jigsaw in their help-seeking journey.

### 3.9 Implementation assumptions

The assumptions outlined in the current program theory model are beyond the direct control of the Jigsaw Live Chat intervention but are likely to influence its implementation success by influencing many elements of the model. Based on findings from the workshop, fourteen key assumptions were identified as necessary conditions for achieving the intended outcomes. These assumptions are outlined in Table 2.

**Table 2:** Jigsaw live chat assumptions.

Assumptions	Brief description
A. Organizational buy-in	For the intervention to be implemented effectively and reach its outcomes, leadership, clinical, and operational staff across the organization must understand what the service offers and believe that it is a beneficial addition to Jigsaw's existing suite of services for young people in Ireland.
B. Community and YP aware of the service	For young people to successfully avail themselves of Jigsaw Live Chat, key stakeholders and gatekeepers such as general practitioners, parents, peers, schools, and colleges must be aware of the service and what the service offers young people.
C. Motivation and readiness to seek support	Young people must be ready and motivated to engage in help-seeking and support provision processes.
D. Flexibility in opening hours	To be perceived as lower-barrier and for young people to attend when they feel they most need support, JLC must offer more flexibility in opening hours.
E. Staff are open to continued training	To facilitate continual training, mental health professionals staffing the service must be open and willing to engage in continual professional development and training.
F. Ongoing maintenance and upgrading of technical system	The technical system upon which the intervention is housed must be upgraded and continually managed to best meet the needs of young people and the service.
G. Young person's capacity to access the service	To access the service, young people must be able to read and write in English, have fine motor skills, moderate digital literacy skills, and be able to seek support when the service is available.
H. Access to a device and reliable Wi-Fi	To access the service, young people must have access to a device through which they can access the internet and have a reliable broadband connection.
I. Minimal wait time	To be perceived as a lower-barrier service than traditional services, young people must experience a minimal wait time to attend the service.
J. Young people do not drop out of the wait queue	To attend the service, the young person must not drop out of the wait queue ahead of entering the chat with a clinician.
K. Clinician is responsive to messages	To contribute to a young person's perception that the service is timely and responsive, clinicians must respond to a young person quickly.
L. Interface is user and youth-friendly	The design of the technical interface and the process through which a young person attends the service must be user-friendly, intuitive, and designed in such a way that is attractive to young people.
M. Youth readiness and motivation to seek further mental health support	Following attending the service, young people may increase their intention to seek further support. To do this, young people must have the readiness and motivation to engage in further help-seeking.
N. Signposting in and out of JLC	To establish itself as a collaborator in the wider system of youth mental health care in Ireland, and to best meet the needs of young people, inward signposting from other services into Jigsaw and outward signposting from Jigsaw to other services must be facilitated.

### 3.10 Using the evaluability assessment: recommendations for measurement and evaluation

This section translates the program theory into a practical evaluation framework, guided by the RE-AIM framework (44, 45). The proposed framework combines outcomes identified from the program theory and builds on this to provide some recommendations for idiographic and data-driven approaches to capture aspects of this framework beyond effectiveness, in line with implementation science approaches to evaluation.

#### 3.10.1 Effectiveness and maintenance (routine and quasi-experimental evaluations)

This section outlines recommended outcome measures suitable for routine use, followed by potential evaluation designs for single-session, chat-based interventions.

Table 3 presents a number of approaches to measuring the core outcomes identified in the program theory. These outcomes provide a foundation for routine monitoring and can support future effectiveness evaluations. However, given the wide variation in why young people access the service, including emotional and informational support, these outcomes may also be captured through idiographic tools. Such tools can complement, or, where appropriate, replace the structured items in Table 3, in routine evaluations where reducing participant burden and detecting individual-level change are priorities. Traditional outcome measures in mental health services often rely on standardized tools designed for consistency across populations. While valuable for comparability, these tools may not fully reflect the immediate, subjective, or diverse needs of young people using single-session chat-based support. In this context, outcome measurement should remain flexible, youth-led, and proportionate to the single session context of the intervention.

**Table 3:** Recommended outcome measurement approaches based on program theory model.

Outcome	Measurement options
Ease of access/ease of use	To assess whether young people perceive the service as easy to access and easy to use, services could post the following single-item questions immediately following the chat session: Did you find [service/intervention] easy to access today? Did you find [service/intervention] easy to use?
Perceived usefulness	With consideration of the many reasons that young people report attending Jigsaw Live Chat, there are several ways to idiographically assess whether the service met the young person's needs. ***Single item questions:*** Single-item questions on perceived usefulness listed below are in line with similar questions asked by chat-based helpline services often offering single-session anonymous support to young people such as LifeLine Chat USA (22) Did you talk about what you wanted to talk about today? Do you feel you got what you needed from today's session? Did you find the chat session useful?
Decreases in psychological distress and overwhelm.	In line with other services such as LifeLine Chat USA (22) examining questions related to psychological distress, or overwhelm could be presented before and after the chat session using a 1–5 scale. For example: On a scale of 1–5 with 1 being not at all distressed and 5 being very distressed, please indicate how distressed you currently feel. ___ (pre-and post-chat session). On a scale of 1–5 with 1 being not at all overwhelmed and 5 being very overwhelmed, please indicate how overwhelmed you currently feel. ____ (pre- and post-chat session).
Help-seeking intentions.	Two simple single-line Likert-style questions could be used to assess young people's intentions to seek further formal and informal support. How likely are you to seek support from another service following your chat session with Jigsaw Live Chat today? (very likely – not at all likely) How likely are you to seek support from another family/friend/trusted adult following your chat session with Jigsaw Live Chat today? (very likely – not at all likely). To enhance usability and engagement, visual analogue scales (VAS) or sliding scale tools may be more appropriate than fixed-response formats.

The following tools represent idiographic and psychometrically validated routine outcome measures that are appropriate for single-session services:

- The Session Wants and Needs Measure (48) is a structured, idiographic tool developed for use within online, single-session chat-based environments. Young people select from a predefined list of common reasons for attendance and are then presented with follow-up questions to rate their progress on the selected concern.
- The Goal-Based Outcome Measure (GBO; 49) is a brief 3-item idiographic tool that allows young people to collaboratively define up to three personal goals with the clinician at the start of the session. The GBO is widely used in youth services (50), and has previously been used in single-session environments (51).

Workshop discussions highlighted the importance of recognizing that not all young people will demonstrate change using standardized psychological measures. Some users may find the session useful in ways that are more subjective or contextual. Idiographic tools can help capture this diversity and ensure the evaluation reflects meaningful indicators of session value, even where traditional change metrics are not applicable. In addition to idiographic tools, workshop discussions also highlighted the potential longer-term role of natural language processing (NLP) and machine learning approaches in supporting outcome measurement in chat-based services. These approaches were discussed as exploratory possibilities rather than immediate evaluation strategies, and would require robust ethical governance, secure and closed data environments, and sufficient organizational capacity to be considered appropriate. Where such conditions are met, NLP-informed approaches may, in principle, support dynamic outcome measurement by:

- Identifying presenting concerns based on chat content (e.g., anxiety, self-harm, relationship difficulties).
- Selecting appropriate measures tailored to the young person's needs, rather than relying on a one-size-fits-all approach.
- Adjusting in real time, capturing both immediate shifts in mental health presentations as well as identifying intentions for future help-seeking.

While these measures are primarily suited to routine monitoring, they may also be integrated into more structured effectiveness evaluations. As outlined above, RCTs are not feasible in the context of single-session, synchronous, chat-based services. However, a quasi-experimental approach may be a viable alternative to evaluation, where other single-session services such as video-based, phone-based, and in-person formats could be employed as natural comparison groups. Such a design could evaluate immediate and sustained mental health outcomes with tools such as the YP-CORE (9), or CORE-10 (40) used alongside the GBO (49), SWAN-OM (48), and assessments of further help-seeking. Additionally, measures of therapeutic alliance [e.g., WAI-SR (52); or SRS; (53)] and subgroup analyses based on demographics can further elucidate for whom, and under what conditions, the intervention is most effective.

#### 3.10.2 Implementation and maintenance: process evaluations

As Jigsaw Live Chat was in the early stages of implementation, a process evaluation was identified as an appropriate next step. This would assess fidelity to the proposed program theory and examine the causal linkages among its components, mechanisms, and outcomes. Specifically, this approach would seek to verify that the intervention is delivered as intended (implementation), while longer-term assessments would address sustainability and continued benefit [maintenance (44)]. To achieve this, a checklist of core intervention components could be developed to evaluate fidelity. This could involve manual coding or via natural language processing (NLP) methods using established lexicons and machine-learning frameworks (54). Interviews with staff and participants could further identify contextual barriers and facilitators, with evaluation activities potentially conducted by both internal staff and external evaluators to balance close working relationships with independent oversight (55).

#### 3.10.3 Evaluations of acceptability (implementation)

To assess intervention acceptability, staff and participants should be surveyed or interviewed using guides based on structured and theory-based assessments such as Sekhon's Theoretical Model of Acceptability (56). This approach would help distinguish between challenges stemming from implementation vs. intervention design and would elucidate key barriers and facilitators to effective delivery, thereby informing further refinements.

#### 3.10.4 Measurement of intervention assumptions (reach)

Workshop discussions and internal data reviews also highlighted key measurement gaps essential for testing the assumptions underpinning the program theory and assessing service reach. These include:

- Self-identified socioeconomic status and social position: To understand whether young people from disadvantaged or minority cultural backgrounds may face barriers to accessing the service, particularly due to digital inequalities.
- Self-identified disability status: To evaluate whether the service effectively reaches young people with disabilities, ensuring inclusiveness and accessibility.
- Geographic location (urban/rural): To determine whether the service is accessible to young people in rural areas, where broadband limitations may pose challenges.
- Device used for service access: Identifying the primary device used to engage with the service could inform interface optimization and accessibility improvements. Knowing whether the device belongs to the young person or someone else could reveal barriers related to privacy, anonymity, and digital access.

To further refine the program theory and ensure evaluation reflects lived experience, consultation with young people who have used single-session live chat support is recommended.

## 4 Discussion

This study applied an iterative, multi-method, participatory evaluability assessment to Jigsaw Live Chat, a national synchronous chat service for young people aged 12–25, in line with updated MRC guidance for evaluating complex interventions (25). This study developed a program theory for a national, single-session synchronous chat service for young people, articulating how the service operates, the outcomes it seeks to achieve, and the contextual conditions under which it is likely to be effective, with the aim of informing feasible, theory-driven evaluation and monitoring. The resulting model outlines specific components and techniques used by staff, the mechanisms of change they are expected to enact, and the outcomes and broader impacts that may follow. These elements were used to generate practical recommendations for evaluation and monitoring, structured using the RE-AIM framework to align evaluability findings with an implementation science approach (44, 45). Collectively, these reflections support theory-driven and implementation-informed evaluation by clarifying what is being evaluated, how change is expected to occur, and how contextual conditions shape evaluability, thereby supporting adaptation and future evaluation of similar services across different settings (57).

### 4.1 Context and implementation

Despite advances in the area and increases in the adoption of live chat services for young people, there is a dearth of robust theory-based evaluations of synchronous live chat interventions across the literature that consider and account for the wider implementation context. Findings from the current study indicate that a range of contextual factors shape how Jigsaw Live Chat is implemented in practice and, in turn, have implications for service reach, sustainability, and the interpretation of evaluation findings. As recognized by staff, nationally, funding of youth mental health services remains a considerable issue. From a small 5% of the overall health budget allocated to mental health, approximately 12% of this is spent on child and adolescent mental health services (CAMHS) in Ireland (2). Staff stressed the need to advocate for support services such as Jigsaw Live Chat to improve buy-in and consistent, stable funding for the service.

Staff also referred to sociocultural perceptions that digital mental health care is “lesser-than” traditional services, highlighting the need for greater understanding among stakeholders of how digital supports can complement face-to-face care within an integrated system. Staff noted that this cross-collaboration was core to the success of Jigsaw Live Chat, perceiving the service as one element of a wider system supporting young people nationally. While the literature recognizes digital support as a core part of integrated youth mental health care globally (58), this perception may not yet be well understood within the local context. Overcoming these perceptions requires a concerted effort from researchers and service providers to better educate stakeholders on the efficacy and benefits of these programs as adjuncts to traditional mental health support.

Staff also outlined how these wider contextual factors could pose barriers to service accessibility, with direct implications for service reach and interpretation of evaluative findings. Specifically, while staff agreed that online synchronous chat counseling services were a lower barrier than traditional “brick and mortar” services, they stressed that this level of accessibility was not necessarily true for all. For instance, broadband connectivity poses barriers to young people living in rural and underserved areas, necessitating the measurement of urban and rural areas of residence in the current service to test this assumption. Beyond connectivity, staff noted that language and literacy demands may also limit access for some groups, particularly given increasing linguistic diversity among young people in Ireland (59, 60). Staff also highlighted that engagement may be challenging for young people with motor impairments, even when voice-to-text tools are available. As such, it was recommended that self-reported disabilities be measured to assess whether the service may unintentionally exclude certain groups of young people.

### 4.2 Therapeutic components, techniques, and mechanisms of change

Identification of key components and techniques used within the current Live Chat intervention are broadly in line with those noted across the literature for chat and text-based mental health interventions. Similar to studies using the Counseling Progress and Depth Instrument (61), a tool to assess therapeutic processes within chat-based interventions (13, 19), problem exploration, goal setting, action planning, and termination of the session were core features of the Jigsaw Live Chat intervention. Further, in line with previous studies (14), expectation management was a core technique used by some clinicians staffing the intervention. Previous literature has reported that young people attending Live Chat services have high expectations of treatment outcomes (14) that may be beyond what can realistically be offered within single-session anonymous support. Managing expectations is key in chat-based mental health services like Jigsaw Live Chat, where support and information provision are central to addressing the wide range of issues young people present. This aligns with findings from Hanley and colleagues (39), highlighting the dual role of such services in offering both emotional support and relevant mental health resources.

The current study provided further insights into the intricacies of intervention components and techniques within the chat-based environment. Namely, the importance of dispelling perceptions of AI was outlined by several staff. This may be unsurprising given the rise of chatbot technologies to support mental health concerns (62). Previous research indicates that mental health and healthcare professionals' perspectives of AI tools to provide care are mixed (63, 64), with evidence also suggesting that users often anticipate poorer conversation quality and lower empathy when using chatbots vs. engaging with a human (65). As a result, there may be a desire within these sessions from both users and providers to clarify how they are staffed. Interestingly, staff noted that they would use colloquial language or deliberately include small imperfections or misspellings to subtly convey that a real person is behind the message. To express emotion and display empathy, they employed explicit descriptors and written affirmations.

Staff stressed the importance of asking clarifying questions to minimize misunderstandings. They also spoke about the need to distinguish helpful pauses from unintended silence. When longer gaps occurred, they would check whether the young person was still present by asking if they were still in the chat. In the absence of visual or vocal cues, staff described communication as more direct and explicit than in face-to-face or video-based work. Within this setting, staff often spoke about the importance of creating a safe and supportive space. They used language, tone, and pacing to help young people feel comfortable and able to discuss their concerns openly. This demonstrates that, even in anonymous and asynchronous contexts, it is possible to create safety, warmth, and connection through language alone. Future research could usefully explore how young people experience this human-led environment, particularly in comparison to emerging AI-based support tools.

Aligned with some of the youth-related activities contained within the theory of change outlined by Hanley et al. (39), the current intervention identified mechanisms of change such as feelings of relief, emotional release, a better understanding of mental health, and access to additional services. Additionally, being validated and understood was important for fostering comfort and openness in discussing concerns with the clinician, which could enhance the development of a strong therapeutic alliance. Again, an in-depth exploration of what this alliance comprised was beyond the scope of the current workshops. However, recent findings have suggested that a combination of promoting agency, autonomy, showing care and empathy, and fostering hope by collaborating on goals was core to the development of a therapeutic alliance in multi-session text-based support with adolescents (66). Staff highlighted these elements as key components and techniques used within the context of the Jigsaw Live Chat intervention. Goal setting is well established as a core facet of therapeutic alliance (67). This appears to hold within a chat-based environment. While the goals identified in single-session contexts may be more immediate and concrete, they nonetheless provide a shared focus for the session. Nonetheless, the extent to which these components are associated with therapeutic alliance in single-session, chat-based contexts remains largely under-researched.

These intervention techniques and components were mapped to the Behavior Change Taxonomy (47). A commonly reported limitation of complex real-world- interventions is the lack of accurate description of their components or “active ingredients” (68, 69). Detailed specification of these active ingredients is crucial for the accurate replication, accurate implementation, and thorough testing of these interventions (47, 68). The current intervention identified ten behavior change techniques. Additional strategies used in the intervention, which are not part of the BCT taxonomy, were also clearly described to support transparency, replication, and future testing or refinement of the program theory.

### 4.3 Outcomes and recommendations for measurement

The outcomes identified in the current study align broadly with those reported by similar chat-based interventions, such Kooth (48), Krisenchat (70), and Lifeline Crisis Chat (22). However, while these studies emphasize accessibility and usability as important service features, they have often prioritized clinical or mental health-related outcomes over these process-oriented measures. This may reflect the focus in digital mental health research on symptom reduction, rather than user experience factors, which are particularly salient for single-session or low-threshold interventions. Given that ease of access and usability are central to the acceptability and effectiveness of services for young people (71), these should be assessed as outcomes in their own right to ensure services remain low-barrier and accessible.

Consistent with findings from other youth-focused chat services, outcomes reported in Jigsaw Live Chat are generally person-specific and vary depending on the reason for attending. This suggests that idiographic or flexible measurement tools are likely more appropriate for capturing change (39, 48). As other studies have noted, young people use these services for a range of reasons, including immediate mental health support (48)**,** which suggests that state-like measures may be useful to capture short-term change. However, many young people also attend for information or lighter-touch support, and the level of psychological distress they experience can vary a lot. As a result, traditional symptom-focused or diagnostic measures may not reflect meaningful change for all users. The outcomes identified in the current study align closely with very recently published international consensus findings, which highlight relational and short-term outcomes such as feeling heard and validated, reduced distress or overwhelm, increased coping, and help-seeker capacity as meaningful indicators of change in youth online chat mental health services (12).

In line with recommendations from other online chat-based services (39), the use of idiographic or self-report measures is recommended within this context. Idiographic measures, such as the Goal Based Outcome Measure, have been widely adopted in youth services and have been employed in Jigsaw's in-person offerings for some time (72). The flexibility of these measures is essential, given the diverse reasons young people attend these services and their capacity to minimize burden for both clinicians and young people. Additionally, goal-based outcome measures have shown effectiveness in single-session support settings (49), and specific measures like the Session Wants and Needs Outcome Measure (SWAN-OM) have recently been developed for youth chat-based contexts (48). In single-session contexts, collaboratively set goals must be both tangible and achievable within the session timeframe, typically focused on immediate relief, problem clarification, or identifying next steps rather than symptom resolution. While conventional measures may currently lack sensitivity to change in single-session contexts, future analyses using larger routine datasets could allow for recalibration of reliable change indices and help determine clinically meaningful thresholds for these brief interventions.

Previous reports have suggested that long-term outcomes of live chat services for some young people may be related to further help-seeking behaviors (70). Further help-seeking intentions were highlighted as a potential immediate outcome of the current service, as Jigsaw Live Chat was recognized as a steppingstone to further support or as part of a collaborative approach to supporting young people.

Findings from the theorizing of the Jigsaw Live Chat intervention indicated a need to integrate additional demographic and service use data to assess the reach, accessibility, and adoption of the service. Specifically, to ensure that anonymous drop-in services remain easy to access and equitable, it is important to collect data on socioeconomic status, disability status, and rural or urban location. While most chat services are now technically accessible across devices, understanding how young people access them, for example via mobile, tablet, or desktop, can help identify potential barriers related to data usage, connectivity, or digital literacy, particularly among those who are digitally excluded.

### 4.4 Strengths and limitations

This study presents one of the first published examples of an evaluability assessment and comprehensive program theory of a synchronous, chat-based mental health intervention for young people. Grounded in updated Medical Research Council (MRC) guidance on complex interventions (25), it provides practical recommendations for assessing implementation and evaluation. Although developed in the Irish context, the program theory provides a transferable framework for routine outcome measurement and the evaluation of process, acceptability, and effectiveness. This foundation supports the adaptation, replication, and refinement of similar services internationally. Interventions such as eHeadspace (Australia), Kids Help Phone (Canada), and Lifeline Crisis Chat (United States) face comparable challenges in demonstrating impact and responding to diverse user needs. By proposing clear and feasible evaluation strategies, this study lays the groundwork for advancing evidence and improving the contribution of digital services to youth mental health.

Another key strength of this study was the participatory approach embedded throughout the evaluability process. Staff were engaged from project inception and co-developed the program theory, building ownership and ensuring service relevance. Multiple avenues for input across the process also enhanced buy-in and supported evaluation capacity among staff. This collaborative structure helped generate not only a robust program theory but also practical and service-specific recommendations for measurement and implementation.

However, the current study should be interpreted with consideration of its limitations. Most notably, young people were not consulted in the design of the program theory. Young people and service users are crucial stakeholders within the context of these interventions, and their perspectives are fundamental to the understanding of how these interventions are theorized to work. Within the current evaluability assessment findings, recommendations for the service included the necessary step of refining the proposed model with a group of young people to ensure its validity and utility. Additionally, it was beyond the current scope of this project to further test and refine this model using a process evaluation, but recommendations for this testing have been outlined.

## 5 Conclusion

The current study applied an iterative, participatory, and theory-driven evaluability assessment to Jigsaw Live Chat, a single-session online synchronous chat-based support for young people across the Republic of Ireland. The resulting co-developed program theory outlines the core components and techniques involved in this intervention, the mechanisms through which change occurs, intended outcomes, and real-world impacts, all shaped by key contextual factors and assumptions relevant to implementation. This process offers a clear framework for evaluating service delivery, outcomes, and implementation. Our findings support the use of low-burden, flexible measurement tools and demonstrate how co-produced, theory-based approaches can enhance the evaluation and design of digital interventions for youth mental health. Crucially, this presents a method and an evaluation approach that can be transferred to similar interventions. Moving forward, refining this program theory model with young people and testing it through real-world implementation will be essential. Embedding this approach more broadly can help digital mental health services demonstrate impact, adapt to complexity, and remain accountable to the young people they aim to support.

## Data Availability

The raw data supporting the conclusions of this article will be made available by the authors, without undue reservation.
