# Supplementary material for: Co-creating a program theory and evaluability assessment for an Irish single-session, synchronous chat-based youth mental health intervention: implications for outcome evaluation
Source: Front Digit Health. 2026 Mar 17;8:1673317. doi: 10.3389/fdgth.2026.1673317 (PMC13036182; doi:10.3389/fdgth.2026.1673317)
Supplement: Supplementary file 1 [file Datasheet1.docx]

**Supplementary Materials**

**Supplement 1:** Program Theory Concepts

| **Program Theory Term** | **Definition** |
| --- | --- |
| Impact | The ultimate outcome, goal, or real-world change that the intervention contributes to but cannot achieve on its own (4). This is likely shaped by the context as much as it is the intervention (7,8). |
| Ceiling of Accountability | Often drawn between outcomes and impact. This ceiling indicates the point at which the intervention no longer accepts responsibility for achieving outcomes and thus does not use indicators to measure them (4). |
| Outcomes | The likely short or long-term effects caused by an intervention’s activities. These can be changes in a psychological state, level of awareness, skill, or behavior (2). |
| Mechanisms of Change | Key to realist evaluation approaches, mechanisms of change are core to understanding why a particular intervention activity leads to an outcome. Inextricably linked to intervention context, these mechanisms explain how within a specific context, an intervention component provides a resource that consciously or unconsciously activates the outcome in the client (1,3). May not always be ‘measurable’ but implicit in the intervention change process (1). For example, emotional expression may bring relief, while therapeutic rapport, facilitated through active listening and empathy, helps young people feel understood. These processes may support greater clarity, self-efficacy, and confidence in help-seeking. |
| Components/Activities | Certain activities or strategies that need to be undertaken to bring about outcomes (4,6,9). |
| Contextual Factors and Implementation Assumptions | External conditions beyond the control of the project that are assumed to be in place for the intervention to function as intended and for intermediate outcomes to be achieved (4,6,9). |

**Supplement 2.** STRiDE Rapid Situational Analysis Checklist.

**
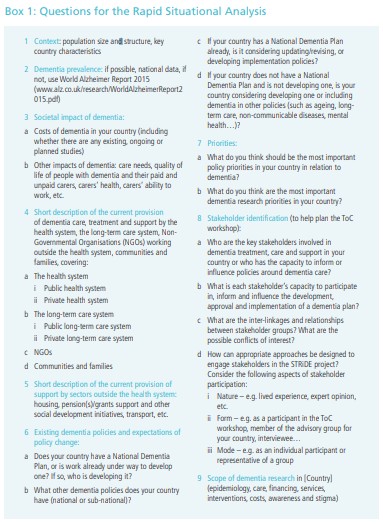
**

**Supplement 3.** Questions posed across workshops.

| **Section** | **Example Questions** | |
| --- | --- | --- |
| **Conceptualization** | What is Jigsaw Live Chat?  What do you think this service is offering to young people?  Who are you trying to reach?  Why do you think young people are attending? |  |
| **Impact** | What do you think the overarching goal or aim of this service is?  [*If you were to look back at this service in 5 years and visualize success, what would this look like?]*  [*Has this changed since the inception of the service?]*  [*Is this likely to change going forward?*] |  |
| **Outcomes** | What changes do you expect to see in young people after attending your service?  What will young people do differently after attending your service?  [*Has this changed since the inception of the service?]*  [*Is this likely to change going forward?*] |  |
| **Mechanisms of Change** | What unconscious/conscious changes are generated in young people following these activities that produce these outcomes? |  |
| **Activities and components** | What are the things that you as a clinician or this service offer/do that make these changes in young people?  What do you generally do at the start of the session?  What happens or what do you do next?  How do you manage risk in the chat session if it arises?  How do you manage lapses or pauses in the chat?  How do you come to a resolution and ensure the young person doesn’t leave in distress?  Is there something you generally do to close the session? |  |
| **Contextual Factors** | What are the specific social, cultural, economic, political, or systemic factors that you think are necessary to be in place for this intervention to succeed? |  |
| **Assumptions** | What resources within Jigsaw are needed for the intervention to function and succeed?  What are the factors within the young person that are necessary for them to successfully engage with the intervention? |  |

***Note.*** Questions presented in square brackets reflect prompts, follow-up, or clarifying questions.

**Supplement 4**. Contextual factors associated with implementation success.

| **Contextual Factors** | **Brief Description** |
| --- | --- |
| 1. Sociocultural perspectives of digital mental health | Community awareness and acknowledgment of digital mental health support as core to the provision of mental health support nationally. |
| 2. Evidence base for digital mental health | The implementation success of Jigsaw Live Chat is dependent upon continued investment into research in digital mental health broadly and specifically, into synchronous chat interventions for youth mental health. This development of a strong evidence base will contribute to increased support for these interventions. |
| 3. Political and Governmental support of digital mental health supports | The implementation of the Jigsaw Live Chat intervention is in line with recommendations from Ireland’s national mental health policy, *Sharing the Vision* (2020), and Ireland’s policy of health reform Sláintecare (2018-2028) which outlines the importance of increased accessibility of services and the importance of evidence-based digital support for mental health. However, it remains crucial that in the face of a changing political landscape, these services remain recognized and prioritized as part of collaborative mental health service provision nationally. |
| 4.Digital mental health policy and implementation roadmap | Currently, in Ireland, there is no digital mental health policy to guide the development and provision of digital services nationally. The development of a guiding policy and clear implementation roadmap could increase recognition and endorsement of digital mental health supports such as Jigsaw Live Chat. |
| 5. Ongoing public sector funding | The continued implementation success of JLC is dependent upon sufficient and ongoing public sector funding of the service. |
| 6. Mental health professional endorsement of digital supports | Support and endorsement from mental health professionals are vital for garnering broader investment in digital chat-based services. Their endorsement may also be influential in decisions regarding staffing for these services. |
| 7. Cross-collaboration and integration with other MH services | JLC operates within a wider system of youth mental health care provision nationally and internationally. For the service to garner implementation success in the long term, JLC must develop relationships and work together with other youth mental health services both nationally and globally. |
| 8. Stakeholder advocacy and promotion | Not only must there be buy-in and support for digital mental health services but for the long-term implementation of JLC, key stakeholders including politicians, service providers, young people, and community members must advocate for the use and benefit of live chat interventions |
| 9. Digital Accessibility and Literacy | Aligned with the objective for European Union member states to achieve internet connectivity goals by 2030, Ireland is presently implementing the *National Broadband Plan*. However, the main challenges to the successful implementation of this rollout are the limited connectivity across rural Ireland, low average broadband speeds, and the increased cost of broadband in comparison to other EU member states (5). Despite these challenges, a substantial portion of Ireland's young population report using the internet, and there is a notable percentage having basic digital literacy skills (70%). The perceived accessibility of live chat services hinges on the effective execution of this rollout and ensuring universal access to high-speed broadband, along with the opportunity for all young individuals to develop proficiency in digital technology usage. |

Supplement 5. Reflexivity Statement.

MT conceptualized the study, led all deductive and inductive elements of the research, and was the primary facilitator across both workshops. At the time of writing, MT is a postdoctoral researcher in the area of youth mental health and digital technologies. MT has worked with Jigsaw since 2019, providing research and evaluation guidance, including while this research was being conducted. MDO’R co-facilitated and took notes during both workshops and collaborated with MT to map the first workshop’s notes onto the program theory framework. MDO’R is a postdoctoral researcher in youth mental health with prior experience supporting Jigsaw’s evaluations and developing program theories. AC co-facilitated the first workshop and, at the time, was employed by Jigsaw as a youth participation coordinator. She is a specialist in participatory research and a qualified clinical health psychologist. JM worked at Jigsaw – The National Centre for Youth Mental Health for many years, serving as Research Manager during the study period and now as Director of Research and Evaluation. His organizational role supported coordination, internal alignment, and final review of the manuscript. AF, an associate professor in youth mental health, supervised the entirety of the project and has collaborated with Jigsaw for many years.

While it is a strength that facilitators had strong working relationships with Jigsaw staff, this may also have shaped workshop dynamics. Familiarity may have fostered trust and richer insights, but could also have introduced bias, constrained critical reflection, or reinforced assumptions aligned with the researchers’ perspectives. At the same time, the research team brought deep knowledge of youth mental health services and a shared commitment to improving outcomes for young people. All facilitators had prior experience in digital mental health research or practice, and the group was deeply engaged with the subject matter. This collective expertise and passion may have enhanced the quality and relevance of the discussions, though it also necessitated ongoing critical reflection throughout the analysis. As such, the reflexive process acknowledged both the risks and benefits of the team’s positionality.

1. Astbury B, Leeuw FL. Unpacking Black Boxes: Mechanisms and Theory Building in Evaluation. Am J Eval. 2010 Sep;1;31(3):363–81.

2. Belcher B, Palenberg M. Outcomes and Impacts of Development Interventions: Toward Conceptual Clarity. Am J Eval. 2018;Dec;39(4):478–95.

3. Dalkin SM, Greenhalgh J, Jones D, Cunningham B, Lhussier M. What’s in a mechanism? Development of a key concept in realist evaluation. Implement Sci. 2015;16;10(1):49.

4. De Silva MJ, Breuer E, Lee L, Asher L, Chowdhary N, Lund C, et al. Theory of Change: a theory-driven approach to enhance the Medical Research Council’s framework for complex interventions. Trials. 2014 Jul 5;15(1):267. doi:10.1186/1745-6215-15-267

5. European Commission. State of Health in the EU Synthesis Report 2023 [Internet]. Luxemburg: European Union; 2023 [cited 2024 Feb 21]. Report No. Available from: https://health.ec.europa.eu/system/files/2023-12/state_2023_synthesis-report_en.pdf doi:doi:10.2875/458883

6. Fridrich A, Jenny GJ, Bauer GF. The Context, Process, and Outcome Evaluation Model for Organisational Health Interventions. BioMed Res Int. 2015;2015(1).

7. Hawe P. Lessons from Complex Interventions to Improve Health. Annu Rev Public Health. 2015 Mar;18;36(1):307–23.

8. Pawson R, Tilley N. An introduction to scientific realist evaluation. In: Evaluation for the 21st century: A handbook. Thousand Oaks, CA, US: Sage Publications, Inc; 1997. p. 405–18.

9. Tsantila F, Coppens E, Witte H, Abdulla K, Amann BL, Arensman E. Developing a framework for evaluation: a Theory of Change for complex workplace mental health interventions. BMC Public Health. 2023 Jun;17;23(1):1171.
